# MIGRATION STATUS, COUNTRY OF RESIDENCE, AND MULTIMORBIDITY AMONG MEXICAN OLDER ADULTS

**DOI:** 10.1093/geroni/igae098.1666

**Published:** 2024-12-31

**Authors:** Nicholas Bishop, Siting Chen, Rafael Samper-Ternent, Ana Quiñones

**Affiliations:** University of Arizona, Tucson, Arizona, United States; OHSU-PSU School of Public Health, Portland, Oregon, United States; The University of Texas Health Houston, Houston, Texas, United States; Oregon Health & Science University, Portland, Oregon, United States

## Abstract

Cross-national data including measures of health and migration status allow investigation of how selection mechanisms may contribute to observed immigrant health advantages. Focusing on Mexican adults aged 50 and older, we drew observations from the 2012 and 2018 waves of the Health and Retirement Study (HRS) and Mexican Health and Aging Study (MHAS) to examine variation in multimorbidity (≥ 2 chronic conditions) across four groups: U.S. born HRS respondents who identify as Mexican (n=674), immigrant HRS respondents who identify as Mexican (n=814), Mexican MHAS respondents who never worked/lived in the U.S. (n=7,833), and Mexican MHAS respondents who worked/lived in the U.S. (n=920). Multimorbidity was defined as the sum of seven self-reported doctor-diagnosed conditions (hypertension, diabetes, cancer, lung disease, heart attack, stroke, and arthritis). Linear regression models were used to predict multimorbidity in 2012 and changes in multimorbidity from 2012–2018 were modeled using an autoregressive approach. Harmonized covariates were measured in 2012 (age, sex, partnership status, BMI, rural residence, # doctor visits in past year). Predicted multimorbidity values for U.S. born Mexicans (y ^=1.65, CI:1.55-1.75) were significantly greater than those for immigrant Mexicans living in the U.S. (y ^=1.35, CI:1.26-1.45), return migrants living in Mexico (y ^=1.35, CI:1.26-1.43), and non-migrants living in Mexico (y ^=1.35, CI:1.30-1.40). Country of residence and migration status were not associated with change in multimorbidity. These results suggest that U.S. born Mexican adults are uniquely affected by multimorbidity and multimorbidity burden among migrants who return to Mexico is similar to immigrant Mexicans who reside in the U.S.

